# Metastatic Intracerebral Chondrosarcoma: Case Report and Literature Review of Endocrine Effects and Management Paradigms

**DOI:** 10.7759/cureus.8417

**Published:** 2020-06-02

**Authors:** Namath S Hussain, Sara H Ahmed

**Affiliations:** 1 Neurological Surgery, Loma Linda University Medical Center, Loma Linda, USA; 2 Endocrinology, Anaheim Regional Medical Center, Anaheim, USA

**Keywords:** chondrosarcoma, intracranial, metastasis, hypercalcemia

## Abstract

The most common underlying diagnosis of intracranial tumor pathology is metastatic disease, followed by primary brain tumors. Chondrosarcomatous metastatic disease of the brain is a rare subtype of this disease process.

The patient presented with right-sided weakness. Her history was significant for femur chondrosarcoma which was resected and treated. Laboratory analysis revealed persistent hypercalcemia and hyperglycemia. MRI of the brain was completed, which revealed a left parietal-occipital lesion with smaller lesions in the left frontal and right parietal lobe.

Multidisciplinary tumor board recommended surgery for lesion resection and pathology. Surgical pathologic diagnosis after lesion resection was metastatic chondrosarcoma. The patient’s preoperative arm and leg weakness improved after surgery.

Our paper delineates this unique case of intracranial spread of femur chondrosarcoma.

## Introduction

Malignant tumors arising from the skeletal system are rare, representing only about 0.5% of all new cancers. Approximately 2100 new cases of malignant bone cancers (sarcomas) occur in the United States each year. The most common type of bone cancer is osteosarcoma. About one-fourth of malignant bone cancers are chondrosarcomas.

The most common cause of neoplastic intracranial mass lesions is metastatic disease, followed by primary brain tumors. Recent studies have shown that brain metastases make up about 49% of intracranial neoplasms while gliomas make up 26%, followed by meningiomas, pituitary adenomas, and schwannomas. The most common metastatic tumors to the central nervous system are lung, breast, prostate, and renal carcinomas. Chondrosarcomatous metastatic disease to the brain is exceedingly rare [1-3]. Isolated cases of metastatic disease from intracranial sources have been observed; however, chondrosarcoma metastasizing to the brain from an extracranial source has not yet been reported in the literature.

## Case presentation

**Figure 1:**
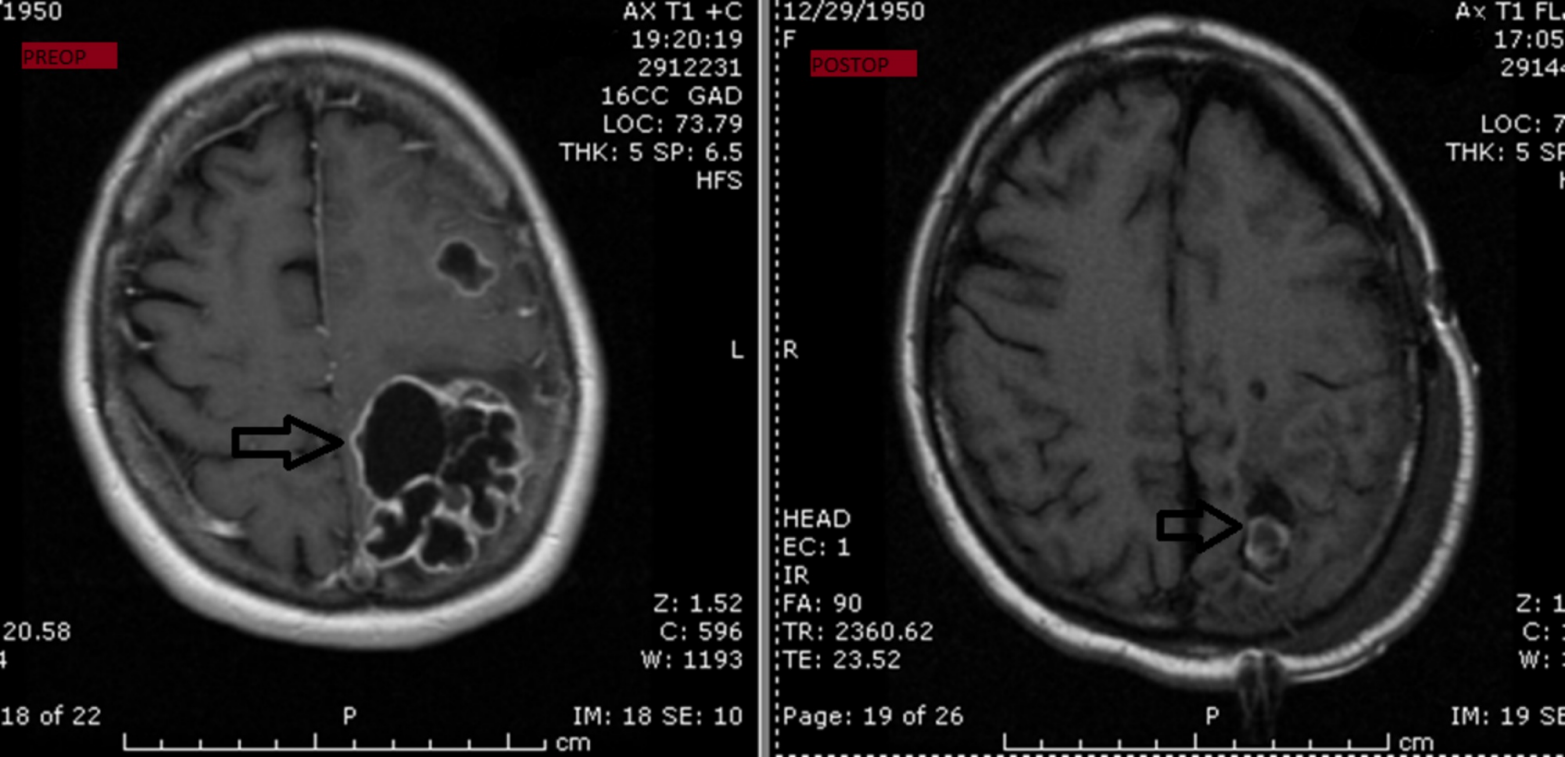
Preoperative and postoperative magnetic resonance imaging of the brain revealing a ring-enhancing mass in the left parieto-occipital lobe.

The patient is a 51-year-old female who came to the emergency department with complaints of right-sided weakness after a recent trip to Mexico. Physical exam revealed 4/5 motor strength in the right arm and leg. An MRI of the brain with and without contrast was obtained, which revealed ring-enhancing lesion in the left parietal-occipital lobe (Figure 1). Smaller lesions were also seen in the left frontal and right parietal intraparenchymal regions. A more in-depth review of her medical history revealed that she had a remote history of left femur chondrosarcoma, which was diagnosed after she had developed severe hip pain in the past. She later developed tumor spread to the lung as well at that time. She also had persistent hypercalcemia and hyperglycemia even after a gross total resection of the femur lesion. She recovered after her femur surgery and was subsequently discharged to home at that time.

**Figure 2:**
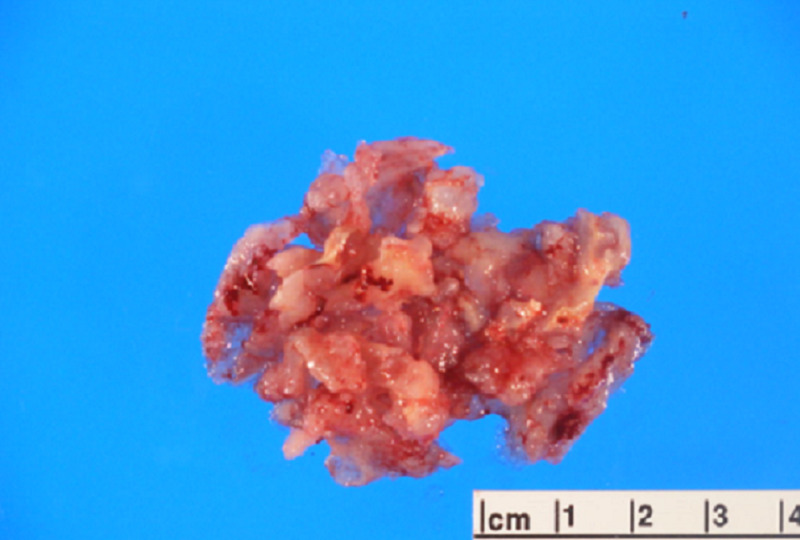
Photograph showing multiple fragments of gray-white, gelatinous multilobulated tissue mass with adherent glistening matrix.

Based on the patient’s previous history and diagnostic studies, our differential diagnosis was intracranial metastasis, primary brain tumor, and neurocysticercosis. Craniotomy for lesion resection and biopsy for pathology was performed, significant for metastatic chondrosarcoma in the resected mass (Figure 2).

**Figure 3:**
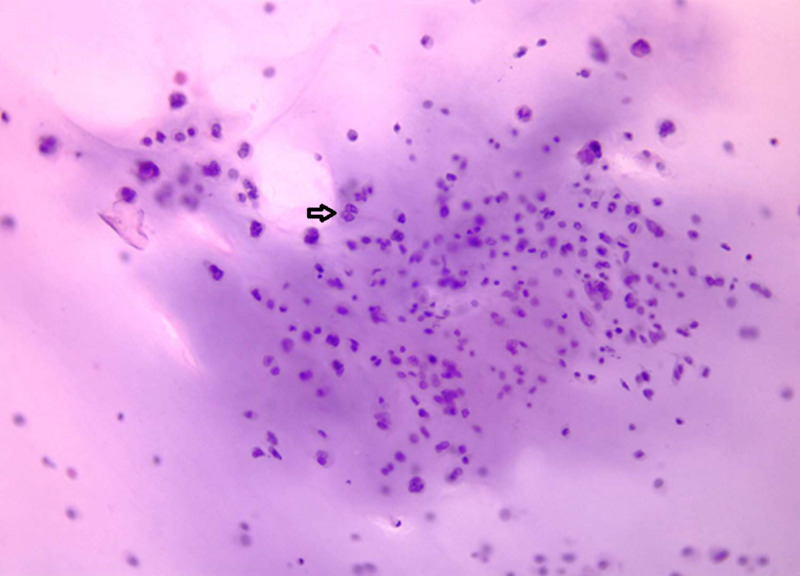
Cytologic smear from gelatinous aspirate showing atypical chondrocytes within a pool of blue mucinous extracellular matrix (papanicolaou stain, original magnification x100).

**Figure 4:**
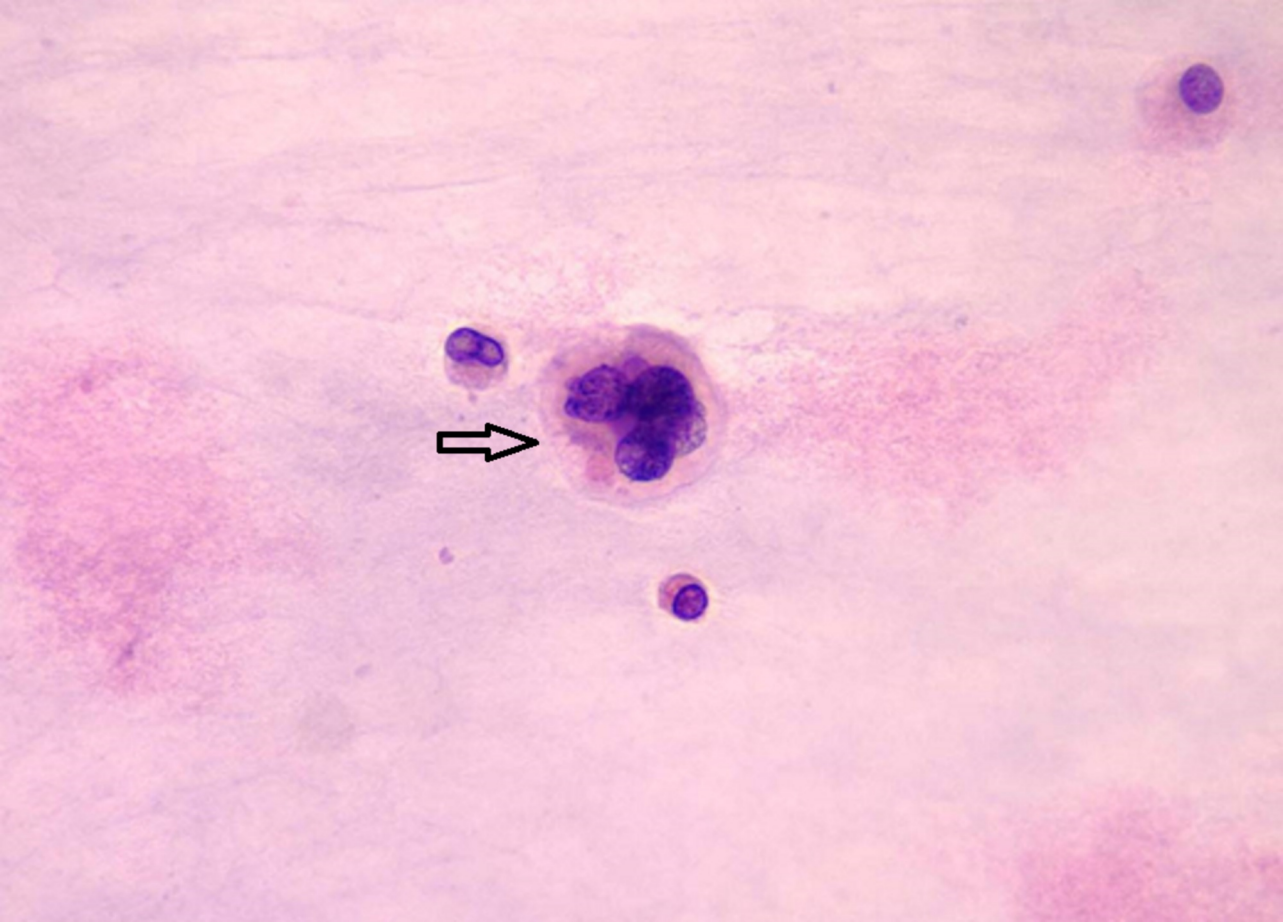
Cytologic smear from gelatinous aspirate showing a large multinucleated chondrocyte with open vesicular chromatin and abundant eosinophilic cytoplasm (papanicolaou stain, original magnification x400).

**Figure 5:**
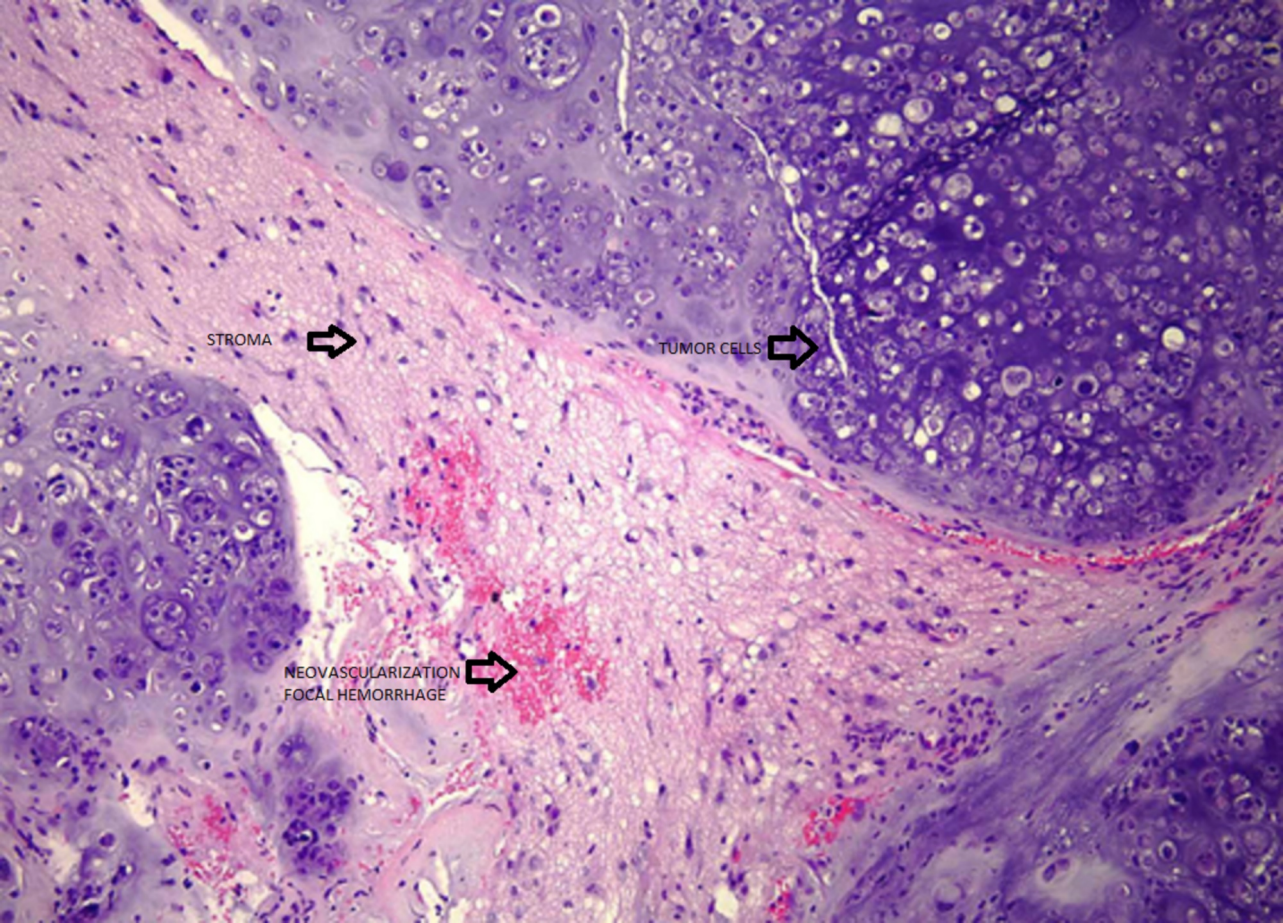
Low power view of chondrosarcoma with nests of tumor cells separated by bands of abundant myxoid stroma. Note neovascularization and focal hemorrhage (hematoxylin-eosin stain, original magnification x100).

**Figure 6:**
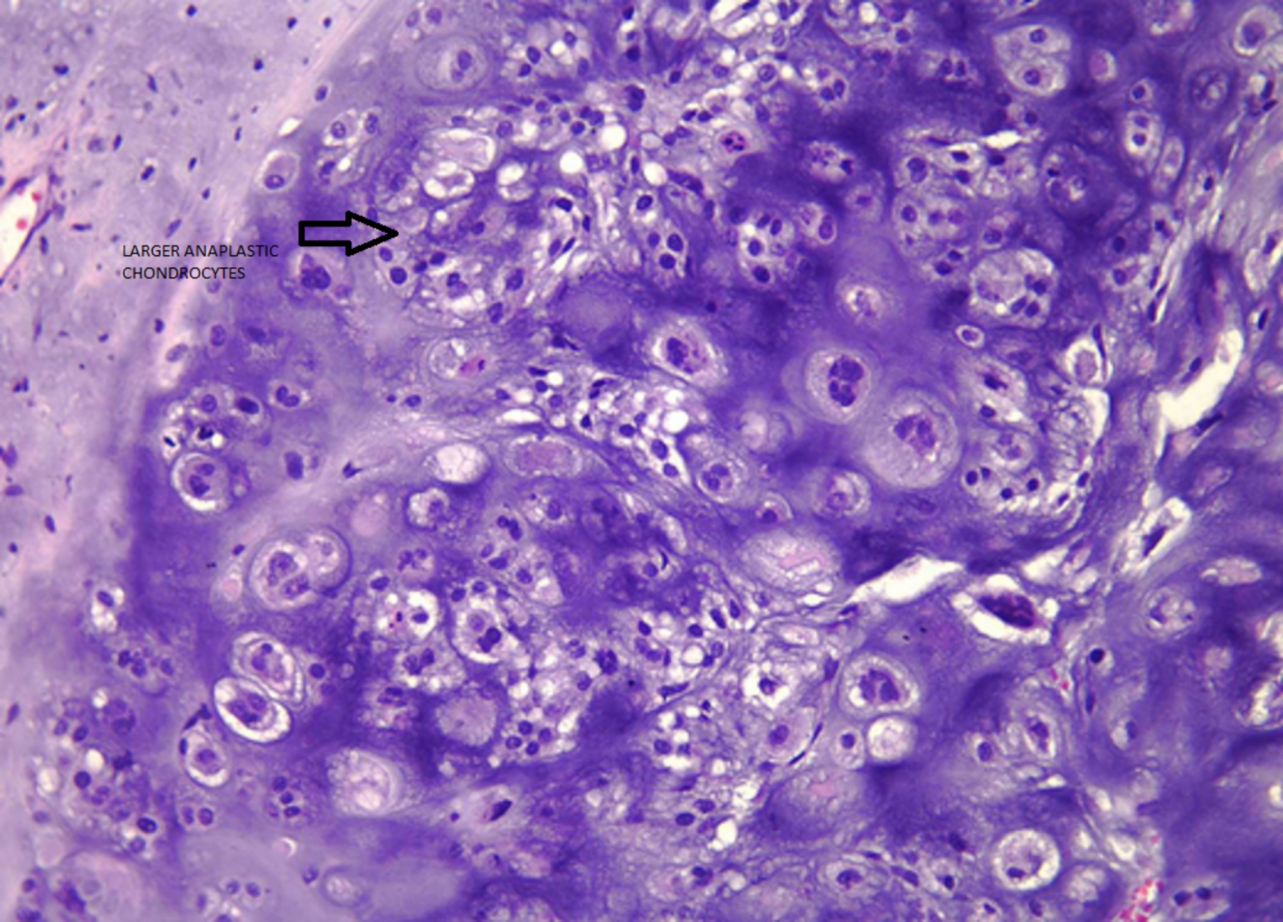
Malignant chondrocytes displaying cellular pleomorphism. Unevenly spaced cells and nests of cells vary in size, shape, and distribution. Larger, more anaplastic chondrocytes exhibit multinucleation, nucleomegaly, and cytoplasmic vacuolization. The extracellular matrix shows a high degree of variability in density (hematoxylin-eosin stain, original magnification x400).

**Figure 7:**
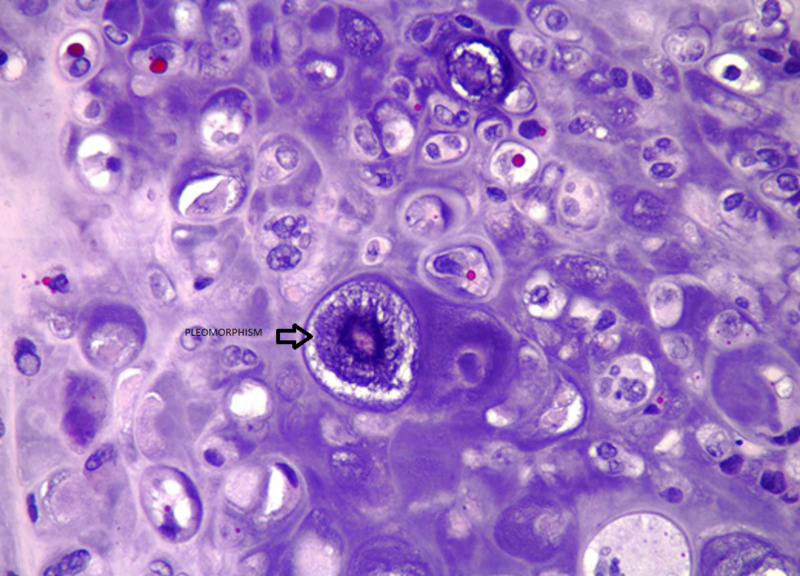
Malignant chondrocytes displaying cellular pleomorphism. Large, anaplastic chondrocytes exhibit bizarre nuclear and cytoplasmic morphology (hematoxylin-eosin stain, original magnification x400).

**Figure 8:**
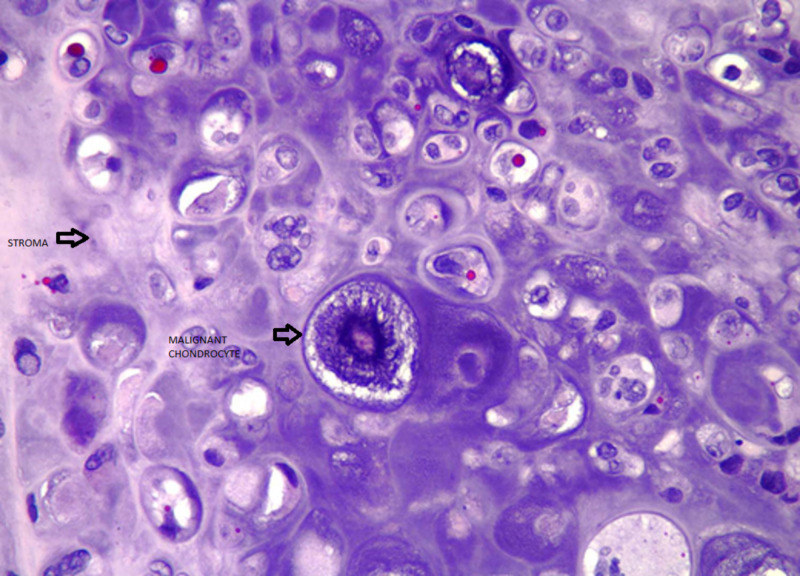
200x magnification revealing hypercellular, malignant chondrocytes in nests and cords displaying frequent mitoses, multinucleation, and nucleomegaly. The extracellular myxoid stroma shows abundant variability in density (hematoxylin-eosin stain, original magnification x200).

Cytology and pathology stain slides were described as sheets of chondrocytes separated by bands of myxoid stroma. The individual cells were anaplastic with mitoses and pleomorphism (Figures 3-8).

After surgical resection, Gliadel local chemotherapy was used. Her weakness improved after surgery. On delayed follow-up, her hyperglycemia and hypercalcemia improved as well.

## Discussion

Chondrosarcomas account for close to one quarter of all primary malignant bone tumors. These lesions typically present in older males. They are located in the femur, tibia, or humerus 50% of the time. Less common locations are the pelvis and spine. They can present as a painful lump or as a pathological fracture of weakened bone.

On imaging these lesions can be a lytic pattern or deep endosteal scalloping and soft-tissue extension. Some have high T2 signal indicative of high-water content and endochondral ossification.

Approximately 50 cases of primary intracranial chondrosarcoma have been reported (Table 1) [1-17]. Intracranial tumors of chondrosarcomatous origin represent less than 0.16% of all primary brain tumors [5]. A comprehensive literature review did not reveal any previously reported case of intracranial metastatic chondrosarcoma from an extracranial source.

**Table 1:** Chondrosarcoma of the brain. GTR: Gross total resection; EB: External beam; STR: Subtotal resection.

Age/Sex	1° or 2°	Location	Treatment	Results	Year	Author
12/M	1°	frontoethmoid bone	GTR	no recurrence	2005	Jroundi et al. [12]
28/F	1°	parafalcine	GTR	no recurrence	2003	Kothary et al. [13]
17/F	1°	frontoparietal cortex	GTR	recurrences at 16 and 19 months	2002	Gonzalez-Lois et al. [8]
69/M	1°	right frontal cortex	GTR	death in 4 weeks due to septic chock	2002	Chaskis et al. [2]
17/F	1°	temporoparietal cortex	GTR	recurrence and death in 3 weeks	2001	Marshman et al. [14]
46/F	1°	frontoparietal cortex	GTR	no recurrence	2000	Oikawa et al. [15]
12/F	1°	falx	GTR, EB radiation (SSagS involved)	progression free	1998	Gerszten et al. [7]
13/F	1°	dura mater	GTR, rad	recurrence in 21 months	1993	Cho et al. [4]
11/F	1°	left parietal cortex	GTR, rad	recurrence and death in 18 months	1992	Chhem et al. [3]
11/M	1°	left occipital	STR	recurrence at 6 months	1979	Rollo et al. [18]
14/F	1°	deep parenchyma	none	death in 8 years	1989	Parker et al. [16]
69/M, 60/M	1°	right temporal bone	GTR	(1) no recurrence at 3 years, (2) no recurrence at 10 months	1985	Adegbite et al. [1]
33/F, 65/M	1°	right posterior fossa, left petrous bone	GTR/rads, GTR	(1) no recurrence at 3 years, (2) no recurrence at 10 months	1985	Hassounah et al. [10]
26/F	1°	left and right frontoparietal	GTR, superior sag sinus resected	intraop death	1980	Heros et al. [11]
59/M	1°	right frontal	STR, rads	no recurrence at 7 months	1980	Richman et al. [17]
28/F	1°	falx	GTR, iodine seeds	recurrence in 8 months	1992	Salcman et al. [19]
20/F, 12/F	1°	Spheno-occip synchondrosis, maxillary sinus	GTR	(1) LTC, (2) death in 3 days	1978	Cianfriglia et al. [5]

Various presentations of primary and metastatic disease from intracranial sources are found in the neurosurgical literature. There have been cases reported on chondrosarcoma causing hyperostosis of the overlying cranium [15]. There have also been reported cases of primary meningeal chondrosarcoma in the literature [15, 16]. In terms of lesion diagnosis, there has been a report of using lamellar inclusions in the rough endoplasmic reticulum as a marker for diagnosis. Other associated diagnostic markers include immunohistochemical positivity for S-100 protein, vimentin, and collagen type II [17].

Accepted treatment methods for primary lesions have usually included total surgical resection; however, there is currently no consensus on adjuvant therapy. Cases utilizing brachytherapy and external beam radiation have been reported with some success [7,16]. Magnetic resonance imaging has come to be accepted as the gold standard in the diagnosis of intracerebral metastatic disease, including chondrosarcoma, with perfusion imaging being the most recent advance in this diagnostic paradigm [13].

A review of papers studying the rationale for using BCNU wafers in the treatment of intracranial metastatic disease reveals that not only are the results promising but also that the mechanism of delivering local chemotherapy directly at the tumor site and obviating the blood-brain barrier can lead to higher local effective concentrations of the drug without systemic toxicity [6, 9]. Randomized, prospective trials are still needed to further elucidate the utility of surgery versus radiosurgery versus local delivery of chemotherapeutic agents to in an effort to bypass the blood-brain barrier.

Hypercalcemia is a common complication of malignancy and occurs in up to 30% of cancer patients. Mild cases of hypercalcemia can cause non-specific symptoms such as lethargy, nausea, and abdominal pain, whereas severe cases can lead to severe dehydration and neurological impairment such as coma. There are several mechanisms that result in hypercalcemia in malignancy. Secretion of parathyroid-related peptide (PTH-rp) by malignant cells, which induces renal calcium absorption, accounts for 80% of cases. Excess osteolytic activity with bone resorption accounts for 30% of cases, and is usually seen in extensive skeletal tumor burden. Increased production of 1,25-dihydroxyvitamin D, which enhances intestinal calcium absorption as well as osteolytic bone resorption, accounts for 1% of cases of malignancy-related hypercalcemia [18].

Hyperglycemia in cancer patients can be attributed to a few proposed mechanisms aside from a known diagnosis of diabetes mellitus. Steroids, which are used in a number of malignancy-related settings such as for cerebral edema from brain metastasis or in several chemotherapy regimens, are a common cause of drug-induced hyperglycemia. In the hospital setting, more than 50% of patients receiving high-dose steroids experience at least one episode of hyperglycemia [19]. Another mechanism for elevated serum glucose levels in critically ill patients is stress-induced hyperglycemia, which results from excess release of counterregulatory hormones, including epinephrine, cortisol, and glucagon. Patient-associated risk factors that have been described in developing both steroid-induced and stress-induced hyperglycemia include older age and higher body mass index [19,20].

## Conclusions

Chondrosarcomatous brain tumors are rare with few primary cases reported in the literature. We report a case of a metastatic intracranial metastasis from an extracranial source, which has not been previously reported. Our case demonstration should help to clarify adjuvant treatment options. Treatment plans should take into account histopathological characteristics of the tumor along with patient functional status, endocrine status, and ability to tolerate adjuvant treatment side-effects. Future studies should address neurocognitive outcomes in addition to recurrence and survival rates with pathological correlation when comparing different treatment algorithms.
